# Intrinsic and extrinsic control impact motivation and outcome sensitivity: the role of anhedonia, stress, and anxiety

**DOI:** 10.1017/S0033291724002022

**Published:** 2024-12-27

**Authors:** Marishka M. Mehta, Grace Butler, Christopher Ahn, Yolanda Irini Whitaker, Keren Bachi, Yael Jacob, Michael Treadway, James W. Murrough, Laurel S. Morris

**Affiliations:** 1 Department of Psychiatry, Icahn School of Medicine at Mount Sinai, New York, NY, USA; 2 Laureate Institute for Brain Research, Tulsa, OK, USA; 3 School of Cyber Studies, The University of Tulsa, Tulsa, OK, USA; 4 Department of Psychology, New York University, New York, NY, USA; 5 Department of Environmental Medicine and Public Health, Icahn School of Medicine at Mount Sinai, New York, NY, USA; 6 BioMedical Engineering and Imaging Institute, Icahn School of Medicine at Mount Sinai, New York, NY, USA; 7 Department of Psychology, Emory University, Atlanta, GA, USA; 8 Freidman Brain Institute, Icahn School of Medicine at Mount Sinai, New York, NY, USA

**Keywords:** anxiety, depression, motivation

## Abstract

**Background.:**

Motivated behaviors vary widely across individuals and are controlled by a range of environmental and intrinsic factors. However, due to a lack of objective measures, the role of intrinsic *v.* extrinsic control of motivation in psychiatric disorders remains poorly understood.

**Methods.:**

We developed a novel multi-factorial behavioral task that separates the distinct contributions of intrinsic *v.* extrinsic control, and determines their influence on motivation and outcome sensitivity in a range of contextual environments. We deployed this task in two independent cohorts (final in-person *N* = 181 and final online *N* = 258), including individuals with and without depression and anxiety disorders.

**Results.:**

There was a significant interaction between group (controls, depression, anxiety) and control-condition (extrinsic, intrinsic) on motivation where participants with depression showed lower extrinsic motivation and participants with anxiety showed higher extrinsic motivation compared to controls, while intrinsic motivation was broadly similar across the groups. There was also a significant group-by-valence (rewards, losses) interaction, where participants with major depressive disorder showed lower motivation to avoid losses, but participants with anxiety showed higher motivation to avoid losses. Finally, there was a double-dissociation with anhedonic symptoms whereby anticipatory anhedonia was associated with reduced extrinsic motivation, whereas consummatory anhedonia was associated with lower sensitivity to outcomes that modulated intrinsic behavior. These findings were robustly replicated in the second independent cohort.

**Conclusions.:**

Together this work demonstrates the effects of intrinsic and extrinsic control on altering motivation and outcome sensitivity, and shows how depression, anhedonia, and anxiety may influence these biases.

## Introduction

Motivation fluctuates widely across individuals and is consistently disrupted in individuals with neuropsychiatric disorders. A broad literature demonstrates that motivation can be driven by multiple factors that can be sub-categorized into two broad camps, encompassing environmental factors, known as ‘extrinsic’ motivation, or self-generated or internal factors, known as ‘intrinsic’ motivation (Morris, Grehl, Rutter, Mehta, & Westwater, 2022; Ryan & Deci, 2020). Motivation itself can be further sub-divided into various processes (Kim, 2013), most broadly into separated domains for action selection and outcome sensitivity (Husain & Roiser, 2018). During action selection, behavior can be selected by the self (intrinsic) or by an external agent (extrinsic). This indicates separations in intrinsic *v.* extrinsic *control* of behavior. Parsing these aspects of control can contribute to our understanding of how they distinctly influence action selection. Traditional effort-based tasks rely on the measurement of extrinsic control to evaluate the utility of effort in value setting and associated motivation drives (Stephens & Krebs, 1986) and other behavioral and computational neuroscience research has largely focused on measuring extrinsic motivation under extrinsic control. Less attention has been paid to intrinsic control. The two control perspectives offer a nuanced insight into effort or cost-utility – *will I pay $10 for a coffee?* and internal value setting – *how much will I pay for a coffee?*

Dysfunction in motivation putatively represents an important transdiagnostic facet of psychiatric symptomology, often classified as distinct psychological constructs, such as apathy in neurological disorders, anhedonia in depression, and exaggerated harm avoidance in anxiety disorders. Anhedonia, for example, a lack of interest, enjoyment, or motivation for previously pleasurable activities, is a core symptom of depression, and is associated with an additional risk of poorer outcomes and morbidity (Ducasse et al., 2018; Leroy, Loas, & Perez-Diaz, 2010; Vinckier, Gourion, & Mouchabac, 2017). Motivational deficits have also been associated with anxiety disorders (Carlton, Sullivan-Toole, Ghane, & Richey, 2020; Enman, Arthur, Ward, Perrine, & Unterwald, 2015; Hopper et al., 2008) and both animal (Hollon, Burgeno, & Phillips, 2015) and human (Dickson & MacLeod, 2004) studies have shown that chronic stress (physical [Kleen, Sitomer, Killeen, & Conrad, 2006] and social [Cabal, Ioanas, Seifritz, & Saab, 2017]) can impair motivational drive (Zalachoras et al., 2022), increasing motivation to avoid perceived harm (Mkrtchian, Aylward, Dayan, Roiser, & Robinson, 2017). This may present as social avoidance or a reduced approach in anticipation or perceived anticipation of social outcomes. The subsequent social disconnectedness is considered a predictor of worse symptoms of anxiety and depression (Santini et al., 2020). While anhedonia and anxiety are linked to problems with overall motivation (Dickson & MacLeod, 2004; Pizzagalli, 2014; Treadway & Zald, 2013), our understanding of the specific contributions of intrinsic *v.* extrinsic control remains limited. This is problematic as it may suggest that whole dimensions of behavior related to intrinsic drive are not being adequately captured using current cognitive assessments. Some work in student populations has suggested that intrinsic motivation may contribute to work ethic (Emmerich & Rigotti, 2017) and depression (Huang, Lv, & Wu, 2016). However, very few cognitive tasks have been developed to specifically measure intrinsic *v.* extrinsic control. Thus, due to a lack of objective measures (for a detailed review, refer to Morris et al., 2022), the role of intrinsic *v.* extrinsic control of motivation in psychiatric disorders remains poorly understood. Parsing psychiatric symptoms more specifically will help to disentangle disorder heterogeneity to improve precision diagnostics and intervention selection.

Intrinsically motivated behaviors are computationally similar to extrinsically motivated behaviors, in that intrinsically motivated behaviors strive to maximize goal attainment and minimize punishment, represented mathematically as value and effort cost functions (Gottlieb, Lopes, & Oudeyer, 2016). Intrinsic motivation is evolutionarily and developmentally critical for exploration and learning, with a prospective orientation (Dayan, 2013; Silvia & Kashdan, 2009), but the definition of intrinsic motivation is often debated (Deci & Ryan, 1980; Kaplan & Oudeyer, 2007). Most prominent theories converge on a definition of intrinsically-motivated behavior as those that are autonomous (Deci & Ryan, 1980; Locke & Latham, 1990), induce a sense of achievement (Chew, Blain, Dolan, & Rutledge, 2021; Deci & Ryan, 1980; Morris et al., 2022) and are enjoyable (Abuhamdeh & Csikszentmihalyi, 2012; Deci & Ryan, 1980; Morris et al., 2022), although the latter two have recently been disputed (Locke & Latham, 1990). Since subjective internal values are difficult to characterize, our understanding of how these values are integrated and used to guide behavior is limited. Paradigms that shift autonomy from extrinsic to intrinsic control, where an individual has autonomy over self-directed goal-setting (Ryan & Deci, 2020), might be useful in addressing intrinsic control of motivated behavior, which is relevant to understanding motivation for both intrinsic and extrinsic outcomes. Here, we focus not on intrinsic reward *per se* but on intrinsic control of behavior.

By building upon well-established work (Chong, Bonnelle, & Husain, 2016; Morris et al., 2020; Treadway, Buckholtz, Schwartzman, Lambert, & Zald, 2009, 2012; Whitton, Treadway, & Pizzagalli, 2015), we have developed an objective cognitive task that separates the distinct contributions of intrinsic *v.* extrinsic control on motivation and outcome sensitivity, in the context of differing valence (e.g. gain *v.* loss). The task was developed to provide important insights into the differing roles of intrinsic *v.* extrinsic control on motivation. We tested this paradigm in two independent cohorts, via in-person and online replication experiments in healthy control (HC) participants and individuals with major depressive disorder (MDD), or an anxiety or stress-related psychiatric disorder (ANX), populations that have shown changes in motivational decision-making. Since consistent evidence has linked depression with reduced motivation during effort-based decision-making tasks (Dickson & MacLeod, 2004; Eshel & Roiser, 2010; Pulcu, Lin, Han, & Browning, 2024; Shankman et al., 2013), we expected that MDD shows a marked reduction in motivation across all domains, both for those under intrinsic and extrinsic control. With regard to symptoms, we also expected that lower intrinsic control of motivated behavior would be associated with the more prospective anticipatory sub-domain of anhedonia. Finally, given the large literature on exaggerated harm-avoidance behaviors in individuals with anxiety disorders (Jiang et al., 2003; Shamblaw, 2019), we expected that higher loss-related motivation would be associated with worse anxiety.

## Methods and materials

### Participants

For the in-person experiment, participants (aged 18–65) were recruited through the Depression and Anxiety Center at the Icahn School of Medicine at Mount Sinai, New York City. Participants were either HC participants, or had a current depressive disorder (MDD), or a current anxiety or stress-related disorder (including generalized anxiety disorder, seasonal affective disorder, post-traumatic stress disorder, or panic; ANX) as their primary psychiatric diagnosis, determined by the Structured Clinical Interview for DSM-V Axis Disorders, performed by a trained rater. Since comorbidity is common, participants in the MDD group were allowed comorbid anxiety if MDD was considered primary, and vice versa for the ANX group, and dimensional clinical scales were used in analyses to assess for variations in all symptom severity regardless of the group, alongside traditional categorical group analyses. Psychoactive medication was allowed in these groups. HC participants were free from current lifetime psychiatric or neurological disorders. Participants with and without a psychiatric diagnosis were excluded if they had an unstable medical issues (including hepatic, renal, gastroenterologic, respiratory, cardiovascular [including ischemic heart disease]; endocrinologic, neurologic – including history of severe head injury, immunologic, or hematologic disease), neurodevelopmental disorder, or drug/alcohol dependence.

For the online experiment, US-based volunteer participants were recruited through the Prolific, Inc. platform. Participants were chosen from a representative sample of the US demographic, with equal split of male/female participants. These online participants were considered a HC volunteer cohort.

All enrolled participants across both studies completed cognitive testing with the Internal–external Motivation Task (IMT), and the same set of self-reported scales assessing dimensions of depression, anhedonia, and stress symptoms, on the same day. Self-reported scales included the Temporal Experience of Pleasure Scale (TEPS) for anhedonia; State-Trait Inventory for Cognitive and Somatic Anxiety (STICSA) for anxiety; and Perceived Stress Scale (PSS) for stress-related symptoms. Symptom scores for each scale were treated as independent measures.

All study procedures were reviewed and approved by the Program for Protection of Human Subjects (PPHS)/Institutional Review Board (IRB) at Icahn School of Medicine at Mount Sinai. Written informed consent was obtained before study participation and all subjects were compensated for their time.

A total of *N* = 190 participants completed the study in-person. Of those, *N* = 9 participants were excluded for not completing the full task (*N* = 6) or making deterministic choices (i.e. making the same button response for every trial, *N* = 3). This left *N* = 181 for analysis, including *N* = 74 HC, *N* = 63 MDD, and *N* = 44 ANX. See Table 1 for in-person participant characteristics.

A total of 326 participants completed the study online. Of those, 258 unique participants successfully completed the full task, with accurate responses to all standardized attention checks and data quality checks. See Table 2 for online participant characteristics.

### Internal–external motivation task

**Figure 1.**
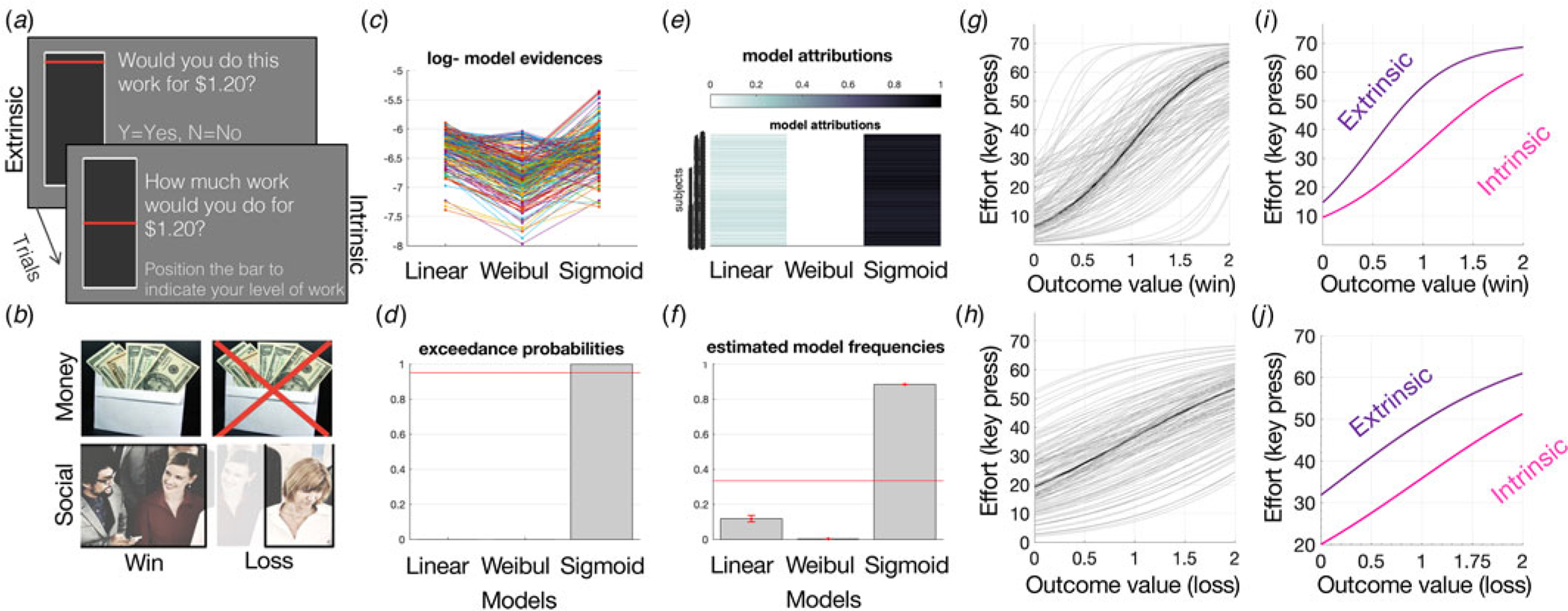
(a) IMT schematic. Each trial requires an effort-based decision for a range of outcomes. In the extrinsic control condition (224 trials), participants must accept or reject the presented amount of effort for a given outcome. In the intrinsic control condition (64 trials), the participant must self-generate the amount of effort they would perform for each outcome. Roughly 20% of trials lead to the effort, all trials are randomly interleaved. (b) Trials led to a range of outcomes, equally divided between: win-money, loss-money, win-social, loss-social, and loss-money. (c–f) Linear, Weibull, and sigmoid models were computed and fit to effort-by-reward discount curves and compared using Bayesian model comparison, illustrating sigmoid as best fit based on (c) log-model evidence, (d) exceedance probabilities, (e) model attributions, and (f) estimated model frequencies. Sigmoid fits for all win (g) and loss (h) money outcomes and separated for intrinsic and extrinsic (i, j) control conditions.

The IMT measures effort-based decision-making, separated by an externally-generated motivation (extrinsic control) condition and an internally-generated motivation (intrinsic control) condition. In the extrinsic control condition, participants were offered a range of outcome levels, paired with a range of effort levels and had to provide a Y (yes) or N (no) response to accept or reject the currently offered amount of effort for the currently offered outcome (Fig. 1). In the intrinsic control condition, participants were offered the same range of outcome levels and indicated the level of effort they were willing to perform for each outcome in an intrinsically-generated manner, by moving a visual bar on the screen (Fig. 1). Note all outcomes were extrinsic thus the task overall tests extrinsic motivation, under the control of either intrinsic or extrinsic choice. The effort was left and right keyboard button presses (up to 70 per trial). The outcomes were either money ($0.25–2.00) or social (25–200 points) rewards or losses, split evenly across 228 trials. Social rewards were operationalized as social points that increase the likelihood of valued group-inclusion paired with positive social facial images in the reward condition or negative social facial images in the loss condition. Gaining more social points in the win condition would increase the likelihood that participants would be included in a highly valued set of research participants, while losing social points in the loss condition would decrease this valued group-inclusion likelihood. Money and social outcome trials were combined into all reward or loss trials. The participants were instructed that the more they worked, the more likely they were to gain rewards in the gain trials or avoid losing rewards in the loss trials, and were more likely to receive a bonus at the end. In reality, all participants received a $2.00 bonus at the end. All trials were randomly mixed, and participants were instructed that 30% of trials were randomly chosen to lead to effort performance (button presses), interleaved, to avoid fatigue. Thus, effort was performed on 30% of randomly selected trials. Outcomes were delivered only at the end of the task and no outcomes or feedback were provided on a trial-by-trail basis as there was no learning involved. Preferences for levels of intrinsic *v.* extrinsic control were therefore tested. The task was programmed using *Psychopy2* and took up to 20 min to complete.

The IMT was adapted for online testing using *Psychopy3*; hosted on their online platform, Pavlovia (https://pavlovia.org/). All task features were identical. The following changes were implemented for online testing. First, the online task training involved more extensive instructions and three multiple-choice questions as a quality control measure to evaluate task understanding. Any incorrect answer led to another session of training for a maximum number of three training sessions before being exited from the study. Second, as an additional attentional check, if the participants responded ‘N’ (no) on 10 trials or more in a row, they received a reminder that by agreeing to more work, they would improve their chances of receiving gain outcomes and avoiding loss outcomes. Third, in the intrinsic condition participants would type in the number of button presses they would be willing to perform instead of moving a visual bar on the screen, without a time limit. Lastly, for the online cohort, after completing the task, all participants received a bonus, unless they responded ‘N’ (no) to >90% of extrinsic trials and entered <5 button presses on average in the intrinsic condition (participants were unaware of these thresholds before participating).

Effort-by-reward discount curves were fit for each trial type (extrinsic-reward, extrinsic-loss, intrinsic-reward, intrinsic-loss) per participant across all cohorts, using three psychometric functions: linear, Weibull, and sigmoid under a mixed-effects framework (see Morris et al., 2020). Parameter estimation and model comparison was performed using the variational Bayes approach to model inversion using the VBA toolbox (https://mbb-team.github.io/VBA-toolbox/) conducted in MATLAB 2019a. Curve fitting within a mixed-effects framework was utilized to reduce the likelihood of outliers for individual parameter estimates. Following one round of model inversion, the population distribution was estimated using individual posterior parameter estimates and inputted as priors for the following round. This was repeated until convergence was met and there was no additional gain in group-level likelihood. Data from all trial types, regardless of intrinsic or extrinsic control condition, were included, meaning that this process was more likely to be conservative with regard to any parameter estimate differences between conditions since individual parameter estimates are shifted toward the group mean.

Curve fitting functions were defined as effort (y) against outcome (x) in the following manner:

1. Linear:
y=mx+c
  where m is the gradient, and c is the y intercept.
2. Sigmoid:
y=c×1/(1+exp(-x-bias)/sigma)
  where bias represents the left–right shift of the curve function (bias against exerting effort, for a given unit of outcome), and sigma is the slope that depicts reward sensitivity (increase in effort related to each unit increase in outcome).
3. Weibull:
$$y=A\times (1-2^{(-x\times L{)}^{s}})$$
  where L is the latency of the function (the minimum outcome required to initiate effort), S is the abruptness (reward sensitivity), and A is the asymptote (effort ceiling for any outcome).

The best fitting curve function that related effort to reward was selected via random-effects Bayesian model comparison that computes the protected exceedance probability as the critical output metric for model comparison that indicates the likelihood that a model is more frequent than others in the comparison set and corrected for the possibility that the observed differences in the model evidences are due to chance (Rigoux, Stephan, Friston, & Daunizeau, 2014) (see Fig. 1). Similar to previous studies (Klein-Flügge, Kennerley, Saraiva, Penny, & Bestmann, 2015; Morris et al., 2020), the sigmoid model was the winning model and was selected for statistical analyses based on the protected exceedance probability and model frequencies (online Supplementary Fig. S1, Fig. 1). Therefore, parameter estimates from the winning sigmoid model (bias and sigma) were derived for each condition for each subject and entered into statistical analyses. The bias parameter was selected as the main parameter of interest as it captures the overall tendency to exert effort for a given outcome, tying closely with motivated behavior.

### Statistics

Repeated measures analyses of variance (ANOVAs) were used to test whether motivation (bias as the dependent variable) was modulated by control *condition* (x2 levels: intrinsic and extrinsic) or *valence* (x2 levels: win and loss), as within subject factors. For the in-person experiment only, *group* (x3 levels: HC, MDD, ANX) was also included as between subjects factor. Second, outcome sensitivity (sigma) was subjected to the same test. Tukey’s post-hoc tests were conducted to further examine any significant effects. This was performed separately for the in-person and online replication cohorts.

Relationships between self-reported symptoms (anticipatory anhedonia, consummatory anhedonia, anxiety, perceived stress) and parameter estimates of motivation (bias) and outcome sensitivity (sigma) for each condition (extrinsic-win, extrinsic-loss, intrinsic-win, intrinsic-loss) were assessed with partial correlation controlling for age, sex, and psychoactive medication status (yes/no). Bonferroni correction was applied scale-wise to correct for multiple comparisons (where Bonferroni-corrected *p* < 0.0125). All results with and without Bonferroni correction are reported.

## Results

Intrinsic and extrinsic control of motivation for rewards and to avoid losses was assessed using two parallel implementations of the IMT (Fig. 1a, b), in MDD (*N* = 63), ANX (*N* = 44), and HC (*N* = 74) tested in-person, and in an online replication sample of presumed HC (*N* = 258). The task measured participants’ willingness to exert effort for outcomes, where the effort levels were either offered (extrinsic control) or self-generated (intrinsic control). Computational modeling of effort-by-outcome discount curves was performed (Fig. 1c–f) within a mixed-effects framework using Bayesian model comparison to derive individual parameter estimates of *bias* as the primary measure of amotivation (Morris et al., 2020) (Fig. 1g–j). Bias provides an estimate of the threshold required for effort initiation, which indicates overall amotivation (where a higher bias value indicates lower motivation). An additional measure, sigma, also referred to as outcome insensitivity, was also computed as a secondary measure. Sigma captures the amount of additional effort exerted for a change in outcome, i.e. the outcome insensitivity, which indicates how much a change in the environment can affect behavior.

### *Effects of intrinsic* v. *extrinsic control on behavior*

**Figure 2.**
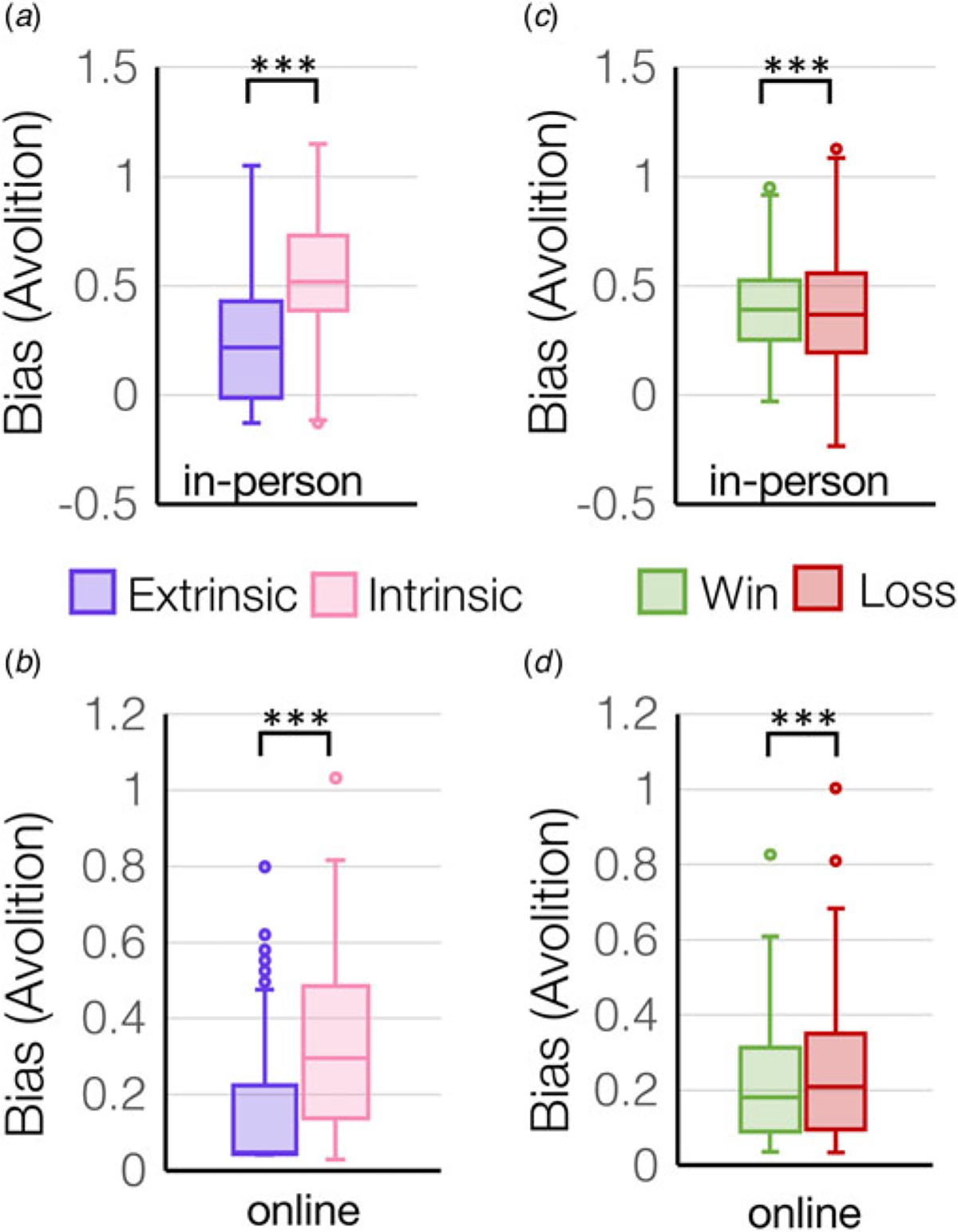
Bias was computed from effort-by-reward discount curves and represents an overall bias away from exerting effort (avolition). Bias is plotted for extrinsic and intrinsic control conditions (a, b) and for win and loss trials (c, d) in the two independent cohorts tested in-person (*N* = 181) or online (*N* = 258). ***p <1 × 10^−10^.

There was a main effect of control (extrinsic control *v.* intrinsic control) on motivation across all participants that was robustly replicated between the in-person cohort (*F*_(1,178)_ = 253.4, *p* = 4.7 × 10^−36^, Fig. 2a) and online cohort (*F*_(1,257)_ = 235.3, *p* = 3.7 × 10^−38^, Fig. 2b), where all participants displayed more extrinsically-controlled motivation compared to intrinsically-controlled motivation. Similarly, participants also displayed higher sensitivity to extrinsically-controlled compared to intrinsically-controlled outcomes in the in-person cohort (*main effect of control condition for sigma*: *F*_(1,178)_ = 44.3, *p* = 3.3 × 10^−10^) (online Supplementary Fig. S1a) and online cohort (*F*_(1,257)_ = 109.2, *p* = 1.6 × 10^−21^). There was also a main effect of valence (rewards *v.* losses) on motivation across all participants that was replicated across both the in-person cohort (*F*_(1,178)=_48.2, *p* = 6.9 × 10^−11^, Fig. 2c) and online cohort (*F*_(1,257)_ = 44.9, *p* = 1.3 × 10^−10^, Fig. 2d), where all participants displayed more motivation for rewards rather than to avoid losses (online Supplementary Fig. S1b). Similarly, participants also displayed higher sensitivity to reward compared to loss outcomes in the in-person cohort (*main effect of valence for sigma: F*_(1,178)_ = 423.7, *p* = 5.9 × 10^−49^). Finally, there was a significant *control*-by-*valence* interaction on motivation in the in-person cohort (*F*_(1,178)_ = 36.9, *p* = 7.3 × 10^−9^), whereby participants showed higher motivation to avoid losses under extrinsic control but not intrinsic control (online Supplementary Fig. S2a). Similarly, in-person participants showed the largest difference in sensitivity to rewards *v.* losses for extrinsically-controlled compared to intrinsically-controlled choices (*control-by-valence interaction for sigma: F*_(1,178)_ = 25.9, *p* = 8.9 × 10^−7^) (online Supplementary Fig. S2b).

### Depression and anxiety distinctly impact extrinsic motivation and intrinsic outcome sensitivity

**Figure 3.**
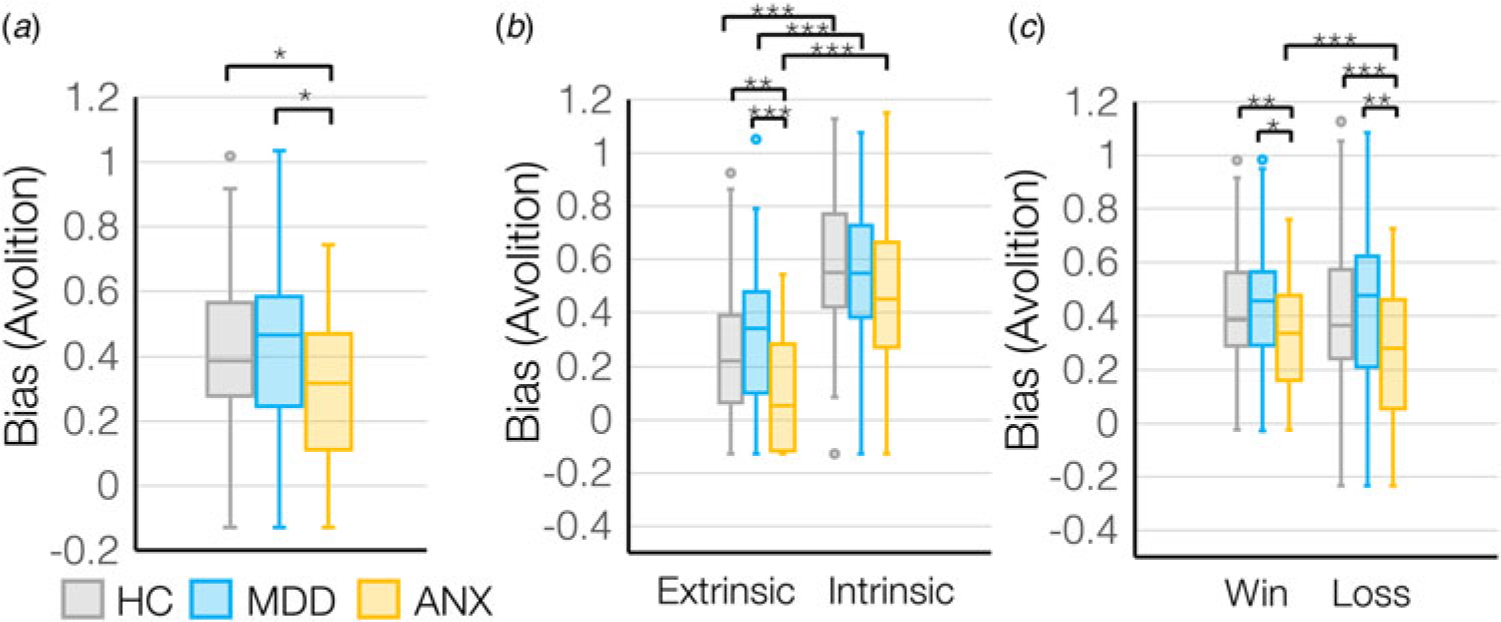
(a) Bias (avolition) values differed across diagnostic groups – HC (*N* = 74), MDD (*N* = 63), ANX (*N* = 44), and based on (b) control condition and (c) win *v.* loss valence for the diagnostic groups. *p < 0.05, **p < 0.01, ***p < 0.001.

There was a main effect of group on motivation (*F*_(2,178)_ = 4.1, *p* = 0.018), where MDD showed the lowest motivation overall whereas ANX showed the highest motivation overall (Fig. 3a). However, outcome sensitivity was lowest in ANX compared to MDD (*main effect of group for sigma*: *F*_(2,178)_ = 3.8, *p* = 0.024) (online Supplementary Fig. S3a), suggesting that ANX show a generalized up-regulation of motivational tendencies without adapting behavior based on a changing environment.

There was a significant interaction between group (HC, MDD, ANX) and control condition (extrinsic control *v.* intrinsic control) on motivation (*F*_(2,178)_ = 4.9, *p* = 0.008) where participants with MDD showed lower extrinsically-controlled motivation and participants with anxiety showed higher extrinsically-controlled motivation compared to HC (Fig. 3b), while intrinsically-controlled motivation was broadly similar across groups. However, again participants with anxiety showed the lowest outcome sensitivity, to intrinsically-controlled outcomes (*group-by-control condition interaction for sigma: F*_(2,178)_ = 6.2, *p* = 0.003) (online Supplementary Fig. S3b), meaning that ANX were insensitive to outcomes that should modulate intrinsically-controlled behavior.

Finally, there was a significant group (HC, MDD, ANX) by valence (rewards *v.* losses) interaction (*F*_(2,178)_ = 5.3, *p* = 0.006), where MDD showed lower motivation to avoid losses than to gain rewards, but ANX showed higher motivation to avoid losses than to gain rewards (Fig. 3c). Interestingly, MDD showed higher sensitivity to losses compared to the ANX (*group-by-valence interaction for sigma: F*_(2,178)_ = 3.5, *p* = 0.033) (online Supplementary Fig. S3c), suggesting that even though the ANX were most motivated to avoid losses, the impact of changes in outcome on changes in effort exertion is not as steep.

### Unique relationships between symptoms, motivation, and outcome sensitivity

**Figure 4.**
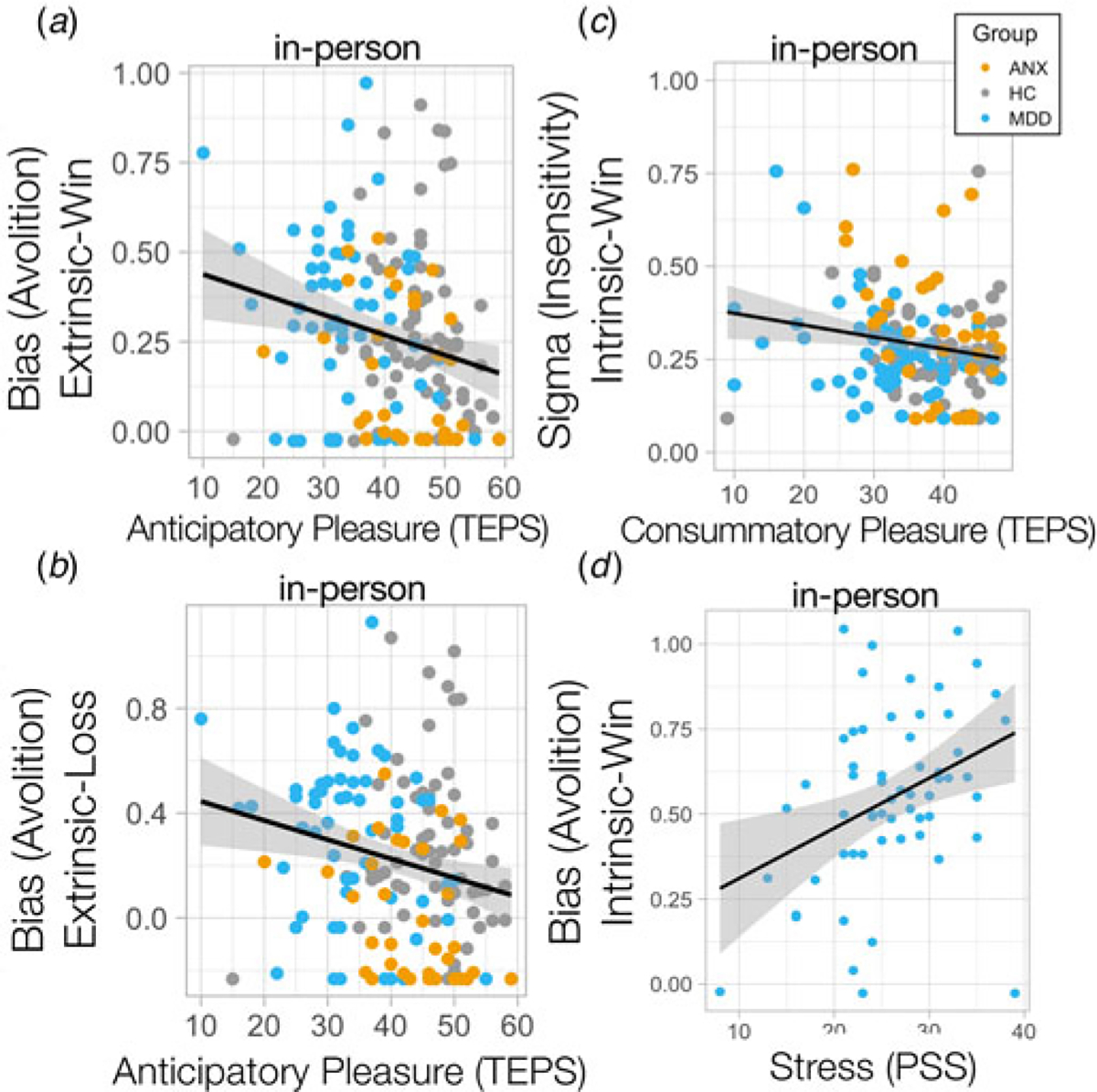
Bias away from exerting effort was associated with anticipatory anhedonia for (a) win and (b) loss outcomes. (c) Insensitivity (sigma) to intrinsically-controlled win outcomes was associated with worse consummatory anhedonia for individuals with MDD and ANX. (d) Stress-related symptoms were associated with lower intrinsic bias (avolition) for win outcomes in MDD.

Lower extrinsically-controlled motivation was dimensionally associated with anticipatory anhedonia across the whole cohort in-person (controlling for age, sex, medications), (extrinsic-win: *R* = −0.2, *p* = 0.015; extrinsic-loss: *R* = −0.213, *p*_Bonferroni_ = 0.009) (Fig. 4a, b), but not consummatory anhedonia (difference in correlations) (extrinsic-win × anticipatory anhedonia *v.* extrinsic-win × consummatory anhedonia: *Z* = −1.39, *p* = 0.081). This was robustly replicated in the online cohort (extrinsic-win: *R* = −0.215, *p*_Bonferroni_ = 0.00051; extrinsic-loss: *R* = −0.162, *p*_Bonferroni_ = 0.009; extrinsic-win × anticipatory anhedonia *v.* extrinsic-win × consummatory anhedonia differences in correlations: *Z* = −1.94, *p* = 0.025) (online Supplementary Fig. S4a, b), specifically linking dimensional anticipatory anhedonia to extrinsically-controlled motivation. On the other hand, lower outcome sensitivity toward intrinsically-controlled outcomes was associated with worse consummatory anhedonia and not anticipatory anhedonia in MDD (intrinsic-win: *R* = −0.363, *p*_Bonferroni_ = 0.005; intrinsic-loss *R* = −0.296, *p* = 0.023; intrinsic-win × anticipatory anhedonia *v.* intrinsic-win × consummatory anhedonia in MDD difference in correlations: *Z* = −1.77, *p* = 0.037) (Fig. 4c) and ANX (intrinsic-win: *R* = −0542, *p*_Bonferroni_ = 0.0009; intrinsic-loss: *R* = −0.590, *p*_Bonferroni_ = 0.0002; intrinsic-win × anticipatory anhedonia *v.* intrinsic-win × consummatory anhedonia in ANX – difference in correlations, *Z* = −3.6, *p* = 0.0002), but not in HC (Fig. 4c). Interestingly, only stress-related symptoms were associated with reduced intrinsically-controlled motivation for rewards in MDD (*R* = 0.323, *p* = 0.021, Fig. 4d), although this did not survive Bonferroni correction for multiple comparisons.

### Complementary behavioral outcomes, internal consistency, and test–retest reliability

The behavioral measures mirrored the computational measures. There were main effects of control condition (*F*_(1,178)_ = 2837.75, *p* = 2.6 × 10^−111^) and valence (*F*_(1,178)_ = 67.24, *p* = 4.6 × 10^−14^) on reaction time, whereby all participants were faster to respond for extrinsic compared to intrinsically-controlled choices and faster for reward outcomes compared to losses (online Supplementary Fig. S5a). There was also a main effect of group (*F*_(2,178)_ = 11.15, *p* = 2.7 × 10^−5^) where MDD were the slowest and ANX were the fastest overall (online Supplementary Fig. S5b). There was a group-by-control condition interaction (*F*_(2,178)_ = 1.91, *p* = 0.021) and a group-by-valence interaction (*F*_(2,178)_ = 3.07, *p* = 0.033) where ANX participants were particularly fast for loss trials and intrinsically-controlled outcomes (online Supplementary Fig. S6). Reliability was measured as internal consistency using Cronbach’s alpha, separately for bias (four items), which indicated excellent internal consistency (α = 0.89) and for sigma (four items), which indicated good internal consistency (α = 0.68). Finally, test–retest reliability of the task was assessed via *N* = 10 participants who repeated the task twice. Although there were apparent marginal individual changes over time, there was good overall stability over time, with no time-dependent differences in measures of motivation or reaction times tested at two separate time points in-person (see online Supplementary Fig. S7) (paired-samples *t* test: extrinsic-win: *p* = 0.38, extrinsic-loss: *p* = 0.31, intrinsic-win: *p* = 0.97, intrinsic-loss: *p* = 0.78).

## Discussion

Using a novel multi-factorial behavioral task across two independent and complementary cohorts, we demonstrated highly consistent results showing that motivation tends to be higher when under extrinsic control compared to intrinsic control, and that sensitivity to outcomes is also higher in the context of extrinsic control relative to intrinsic control, across all cohorts. MDD showed reduced extrinsically-controlled motivation compared to participants with anxiety, who showed the highest extrinsically-controlled motivation, particularly to avoid losses, compared to HC. We further report a double-dissociation in symptoms where while anticipatory anhedonia was specifically associated with reduced extrinsically-controlled motivation, consummatory anhedonia was specifically associated with reduced sensitivity to outcomes that influence intrinsically-controlled motivated behavior. Together this work demonstrates how intrinsic and extrinsic control can alter motivation and sensitivity, and shows how depression and anxiety influence these biases.

The exact definition of intrinsic motivation is hotly debated (Locke & Latham, 1990; Oudeyer & Kaplan, 2007; Ryan & Deci, 2020). A long-standing theory of intrinsic motivation defines it as behaviors that are autonomous, generate a sense of competence or skill mastery, and foster relatedness or social connection (Ryan & Deci, 2020). A more recent theory that is in direct conflict with this, defines intrinsic motivation as taking pleasure in an action or activity, separate from any sense of achievement, mastery or social confirmation, or any gain/loss (Locke & Schattke, 2019). Since the current task uses extrinsic outcomes only, we focus instead on the intrinsic or extrinsic nature of control, which is an important aspect of intrinsic motivation that may have been previously overlooked. This work suggests that intrinsic motivation can arguably be sub-divided into two broad processes: sensitivity to intrinsic outcomes (such as pleasure in partaking in an activity), and intrinsic control of behavior. In the context of these differing theories, we highlight here how a simple shift of autonomous control, from extrinsic to intrinsic, can profoundly influence motivation and outcome sensitivity, that is independent from achievement, and is consistent across a range of differing contexts of social relatedness or monetary gain or loss (Fang, Treadway, & Hofmann, 2017; Morris et al., 2022).

All individuals regardless of group consistently demonstrated higher extrinsically-controlled motivation compared to intrinsically-controlled motivation in both studies, despite the use of the exact same offers of rewards and losses. This might be explained by the fact that the intrinsically-controlled motivation condition required an initial goal-setting processes, whereas the extrinsically-controlled condition did not, thus adding a second cognitively effortful decision-making process during the task, in order to search for their individual indifference point. The level of autonomy might be similar in both conditions as the participant ultimately decides whether they will agree to the offer and at what level, but the amount of agency required in determining the level might differ. The conditions also differ with respect to the offers being categorical (yes/no offer, extrinsic) *v.* dimensional (dimensional range offered – intrinsic), which could require further cognitive effort in the latter case. The observed results therefore can be explained within the prospect theory framework, whereby in the extrinsic control condition the reference point is pre-set, while in the intrinsically-controlled condition, the reference point must be deliberated and selected internally. This also explains the largely higher reaction times required in intrinsically-controlled compared to extrinsically-controlled condition, although it does not explain lower sensitivity in intrinsically-controlled compared to extrinsically-controlled conditions. Perhaps when the reference point is pre-set (extrinsic), it removes an amount of effortful deliberation required and diminishes the valuation of the outcome in terms of its impact on behavioral change. Another explanation along the same lines is that research participants by nature can be considered somewhat already-motivated and self-selected for agreeableness. Follow-up work exploring personality traits such as agreeableness might help explain this large difference in motivated behavior in the extrinsic control condition (i.e. more ‘yes’ responses to offers).

MDD showed lower extrinsically-controlled motivation compared to those with ANX, especially to avoid losses, associated with worse symptoms of anticipatory anhedonia. This indicates a difficulty in avoiding negative outcomes that are extrinsically controlled, in line with a wealth of literature demonstrating reduced motivation and reward sensitivity in depression (Dickson & MacLeod, 2004; Eshel & Roiser, 2010; Pulcu et al., 2024; Shankman et al., 2013), and provides further explanation of observed deleterious behaviors in the real world where negative outcomes are not avoided. On the other hand, and contrary to our expectations, intrinsically-controlled motivation seemed to be broadly intact in depression. This lack of difference in intrinsically-controlled motivation in depression might explain some recent inconsistencies in the literature (Moutoussis et al., 2018; Vergara & Roberts, 2011) and highlight a target for intervention that could enhance the already-present intrinsically-controlled motivation, steering behavioral activation toward behaviors that are intrinsically-controlled. This work also demonstrated that those participants with worse consummatory anhedonia had reduced sensitivity to outcomes that modulated intrinsically-controlled behaviors, meaning that they were not using environmental information to update intrinsically-motivated behaviors appropriately. This suggests that by up-regulating sensitivity to outcomes obtained specifically via intrinsically-controlled behaviors individuals may demonstrate improvements in consummatory anhedonia.

Individuals with anxiety disorders showed the highest motivation overall (Bishop & Gagne, 2018), but the lowest sensitivity to intrinsically-controlled outcomes, as suggested by the sigma parameter. Within the sigmoid curve fit model, a higher sigma describes a more flat, less steep discount curve meaning that incremental increases in outcome magnitude lead to small changes in effort exertion. In contrast, a lower sigma describes a steep discount curve where incremental changes in outcome magnitude govern larger increases in effort expenditure. Since individuals with anxiety disorders had higher sigma levels compared to MDD (but not HC), this suggests that the relationship between outcome magnitude change and effort exertion was more weak. Since individuals with anxiety disorders had the highest levels of motivation overall, this potentially suggests a more generalized non-specific increase in motivation that is divorced from the outcome information that should be used to guide behavior. These results may reflect the harm-avoidance tendencies affiliated with high trait anxiety (Jiang et al., 2003) and stress exposure (Shamblaw, 2019) governed by dysregulation of salience appraisal (Neumann, Glue, & Linscott, 2021). Often the resultant observed behavior is goal-directed toward avoiding negative outcomes. Another interpretation of a higher sigma parameter (flatter curve) is that these functions are more linear compared to a lower sigma (steeper curve) which might represent more binary decision-making where around a certain magnitude of outcome there is more of a switch from low to high effort exertion. Within this interpretation, individuals with anxiety disorders here would show more linear relationships between changes in outcome and exertion of effort, compared to MDD who show more binary decision-making.

Regarding limitations, we did not observe an expected effect of valence on motivation, whereby losses ‘loom larger’ than gain, and would be expected to be avoided with higher motivation than gains. This might be explained by an incongruent ‘approach-avoid’ aspect of the task where participants must approach, or agree to the exertion of effort for, losses, rather than avoid them. Further work teasing apart these approach/avoid features of the task and the cognitive effort required to complete the task will be required to fully explain these behavioral phenomena. Second, the task distinguishes the nature of control conditions by operationalizing choice as categorical (extrinsic) *v.* dimensional (intrinsic). The dimensional framing of intrinsic control allows for the examination of self-generated aspect of intrinsic motivation. The self-generated effort was hypothesized to reflect the subjective value of the task, which may encompass the reward value and task experience to varying degrees. However, this approach does not fully capture all aspects of intrinsic control defined above (Locke & Schattke, 2019; Ryan & Deci, 2020). This limited view of intrinsic control may explain the lack of differences in intrinsically-controlled motivation in individuals with depression. Third, we did not measure self-reported intrinsic/extrinsic motivation to assess relationships with behavioral measures. This should be the subject of future work. Fourth, test–retest stability was only measured in a small sample of participants. Follow-up multi-session testing of this task will be required to establish test–retest reliability. Last, the current task used a maximum of 70 button presses which might have been considered low and led to a ceiling effect in motivation whereby some individuals who were highly motivated reached their maximum at low levels of outcome. While we did exclude datasets with deterministic values (i.e. all ‘yes’ or maximum responses on every trial), further work that tailors the amount of effort to each individual might better capture individual differences in motivation without ceiling effects.

## Supplementary Material

Supplementary material

## Figures and Tables

**Table 1.** Characteristics of the participants from the in-person sample

	HC	MDD	ANX	*p* value
*N*	74	63	44	
Age (mean ± s.d.)	31.5 ± 9.5	32.7 ± 10.6	33.0 ± 10.5	0.724^a^
Gender (*N*, %)				
Females	41 (55.4)	35 (57.4)	32 (72.7)	0.207^b^
Ethnicity (*N*, %)				
Hispanic/Latino	9 (12.2)	15 (24.6)	9 (10.5)	0.063^b^
Race (*N*, %)				0.005^b^
White/Caucasian	28 (37.8)	39 (63.9)	20 (45.5)		
Black/African American	16 (21.6)	8 (13.1)	3 (6.8)		
Asian	24 (32.4)	5 (8.2)	10 (22.7)		
American Indian/Alaskan	1 (1.4)	0 (0)	1 (2.3)		
More than one race	3 (4.1)	7 (11.5)	7 (15.9)		
Unknown/do not wish to disclose	2 (2.7)	2 (3.3)	3 (6.8)		
Employment status (*N*, %) (includes part-time and full time)				
Employed	48 (64.9)	35 (57.4)	29 (65.9)	0.583^b^
Education (*N*, %) (includes those who have completed/are completing college, and those who have completed/are completing graduate school)				
Higher education	70 (94.6)	52 (85.2)	36 (81.8)	0.076^b^
Marital status (*N*, %) (includes those who have never been married or are divorced, separated, or annulled)				
Married	15 (20.3)	16 (26.2)	11 (25)	0.599^b^
Not married	59 (79.7)	44 (72.1)	33 (75)	
Medication				
Psychoactive medication (*N*, %)	1(1)	32 (51)	19 (43)	
Depression				
*N*	73	57	44	
Depression Severity	1.55 ± 1.84	13.86 ± 4.65	8.27 ± 4.70	<0.001^a^
*N*	67	58	34	
Anhedonia (Anticipatory)	46.52 ± 7.18	34.12 ± 8.61	43.18 ± 7.74	<0.001^a^
Anhedonia (Consummatory)	38.60 ± 6.96	32.45 ± 8.72	37.94 ± 6.78	<0.001^a^
Anxiety and stress				
*N*	52	38	38	
Anxiety (cognitive)	11.17 ± 2.21	25.32 ± 6.44	24.84 ± 7.22	<0.001^a^
*N*	51	38	38	
Anxiety (somatic)	11.82 ± 1.51	20.55 ± 7.59	18.79 ± 6.37	<0.001^a^
*N*	69	58	39	
Perceived stress	9.55 ± 5.24	26.16 ± 6.24	22.10 ± 7.22	<0.001^a^

Depression severity, QIDS total score; Anhedonia (anticipatory), TEPS anticipatory score; Anhedonia (consummatory), TEPS consummatory score; Anxiety (cognitive), STICSA cognitive sub-score; Anxiety (somatic), STICSA somatic sub-score; Perceived stress, PSS score.

a One-way ANOVAs.

b χ^2^ test.

**Table 2.** Characteristics of the participants from the online sample


*N*	258
Age (mean ± s.d.)	32.86 ± 6.55
Gender, female (frequency, %)	135 (52.33%)
Hispanic ethnicity (frequency, %)	19 (7.36%)
Race, white (frequency, %)	204 (79.07%)
Employment status, part-time and full time (frequency, %)	201 (77.91%)
Education (frequency, %) (includes those who have completed some college, graduated from 4-year college, and have completed/completing an advanced degree)	193 (74.80%)
Marital status, married (frequency, %)	111 (43.02%)
Depression	
Depression Severity	3.5 ± 6.22
Anhedonia (anticipatory)	42.57 ± 7.87
Anhedonia (consummatory)	37.19 ± 6.21
Anxiety and stress	
Anxiety (cognitive)	19.65 ± 8.21
Anxiety (somatic)	16.52 ± 6.09
Perceived stress	17.29 ± 9.20

Depression severity, QIDS total score; Anhedonia (anticipatory), TEPS anticipatory score; Anhedonia (consummatory), TEPS consummatory score; Anxiety (cognitive), STICSA cognitive sub-score; Anxiety (somatic), STICSA somatic sub-score; Perceived stress, PSS score.

## Abbreviations:

ANX: anxiety or stress-related psychiatric disorder
HC: healthy control
IMT: Internal–external Motivation Task
MDD: major depressive disorder
PSS: Perceived Stress Scale
STICSA: State-Trait Inventory for Cognitive and Somatic Anxiety
TEPS: Temporal Experience of Pleasure Scale
