# Supplementary material for: Intrinsic and extrinsic control impact motivation and outcome sensitivity: the role of anhedonia, stress, and anxiety
Source: Psychol Med. Author manuscript; Available in PMC 2026 Apr 10. (PMC11769897; doi:10.1017/S0033291724002022)
Supplement: Supplementary material [file NIHMS2045865-supplement-Supplementary_material.pdf]

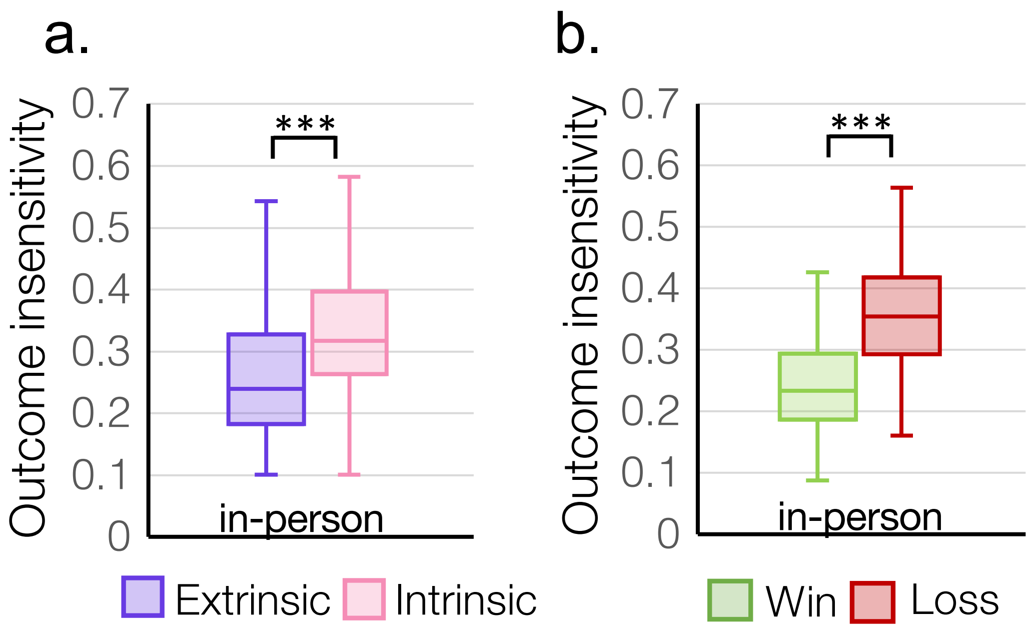

Supplementary Figure 1. Outcome insensitivity values (sigma) were assessed across (a) control and (b) valence dimensions in the in-person sample.
***$p<1x10^{-10}$

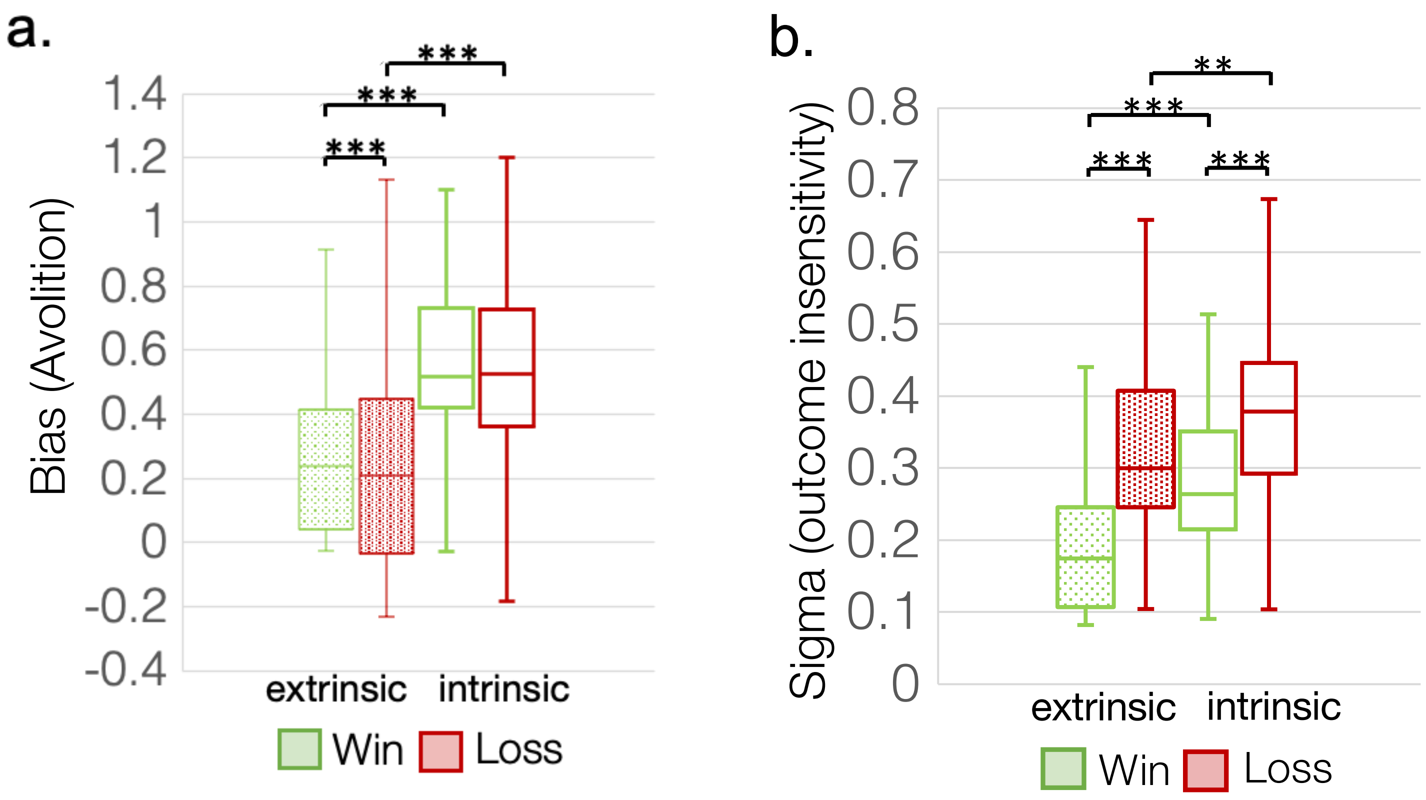

Supplementary Figure 2. A significant control-by-valence interaction was observed for (a) bias (avolition), and (b) sigma (outcome insensitivity) in the in-person cohort. **p=0.00002 ***p<1x10-17

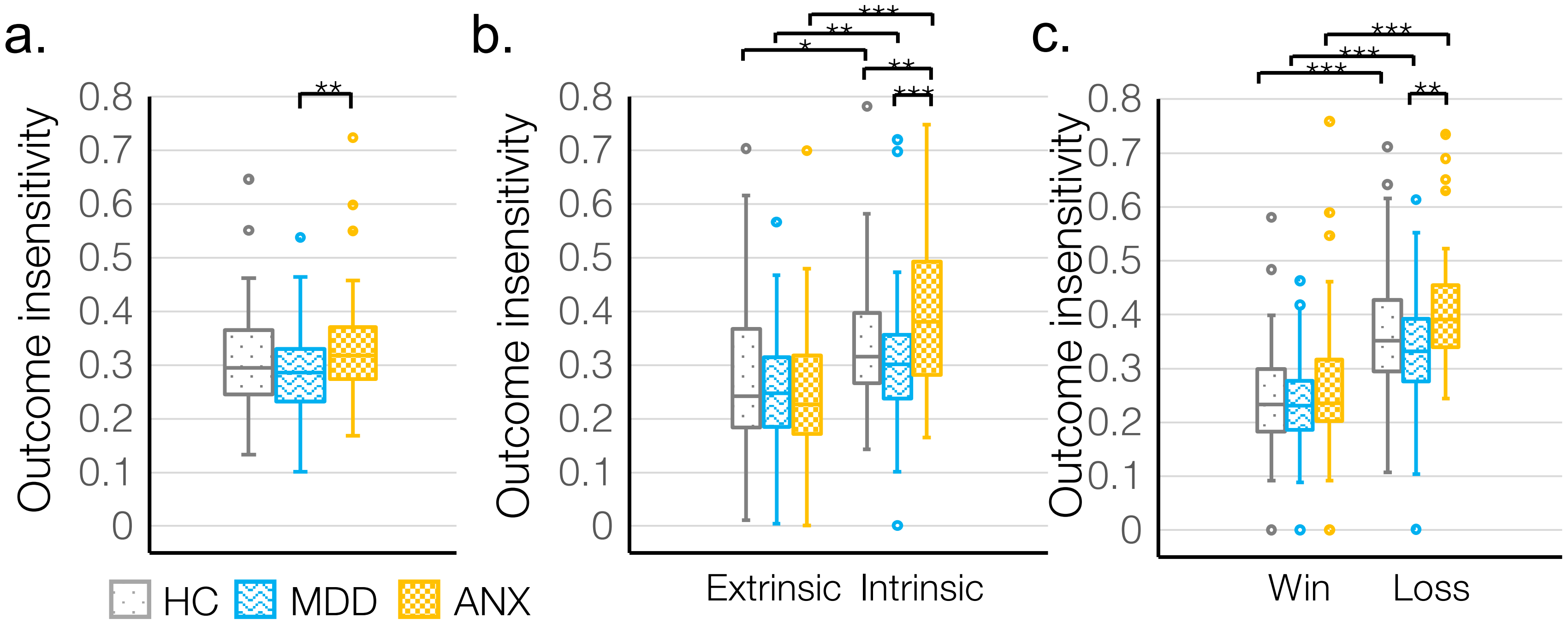

Supplementary Figure 3. (a) Sigma (outcome insensitivity) values differed across diagnostic groups – Healthy Controls, HC (N=74), Major Depressive Disorder, MDD (N=63), anxiety and stress-related disorders, ANX (N=44). Sigma also varied across (b) control condition and (c) win versus loss valence for the diagnostic groups. *p=0.01 **p<0.007 ***p<0.0007

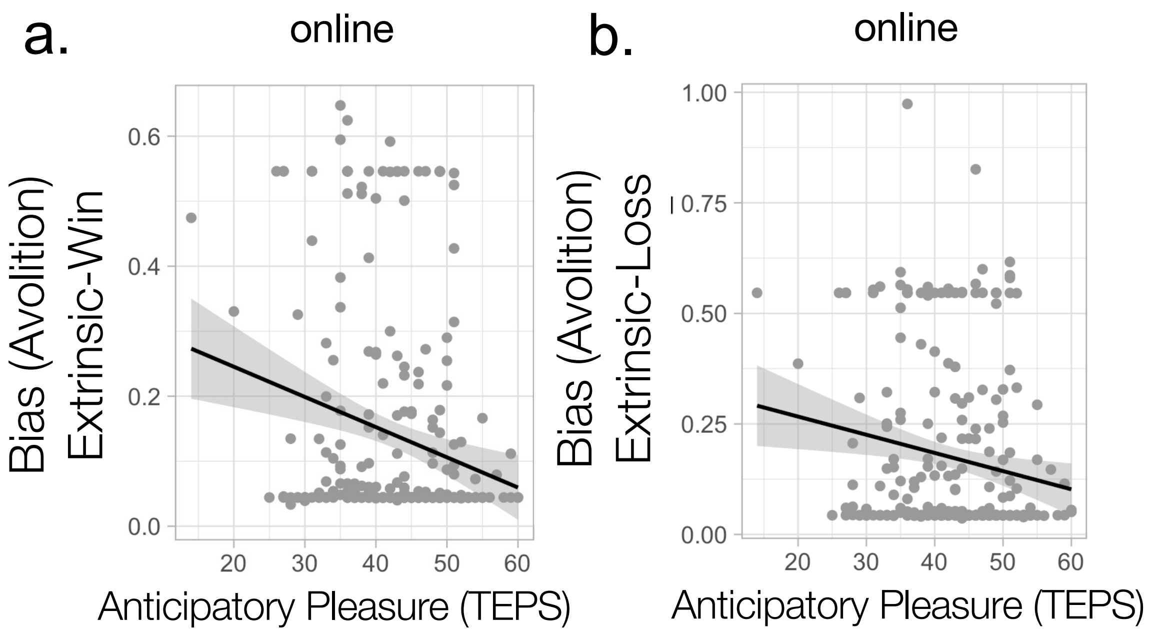

Supplementary Figure 4. Bias (avolition) was associated with anticipatory anhedonia for (a) win and (b) loss outcomes in the online cohort.

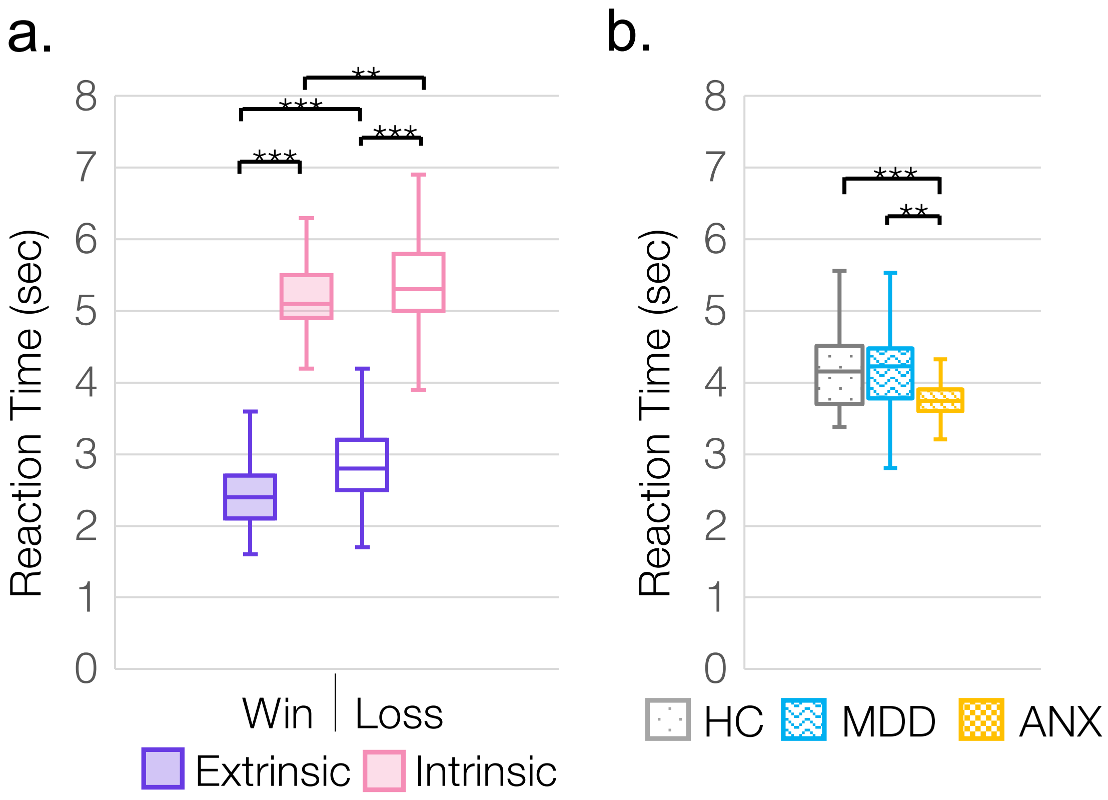

Supplementary Figure 5. (a) Participants responded faster in extrinsic compared to intrinsic control trials and faster for win compared to loss outcomes. (b) Individuals in the anxiety and stress-related disorders (ANX) group were fastest and those in Major Depressive Disorder (MDD) were the slowest, compared to healthy controls (HC). $^{**}p<1\text{x}10^{-5}$ $^{***}p<1\text{x}10^{-6}$

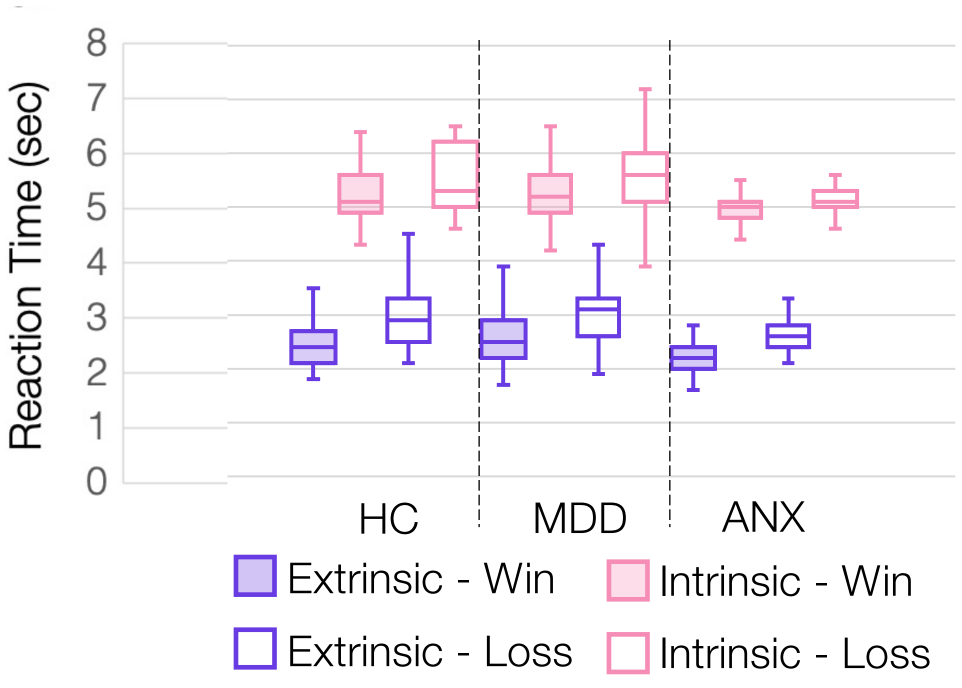

Supplementary Figure 6. Group-by-valence interaction for reaction times. Individuals with ANX were particularly fast for loss trials and intrinsically-controlled outcomes.

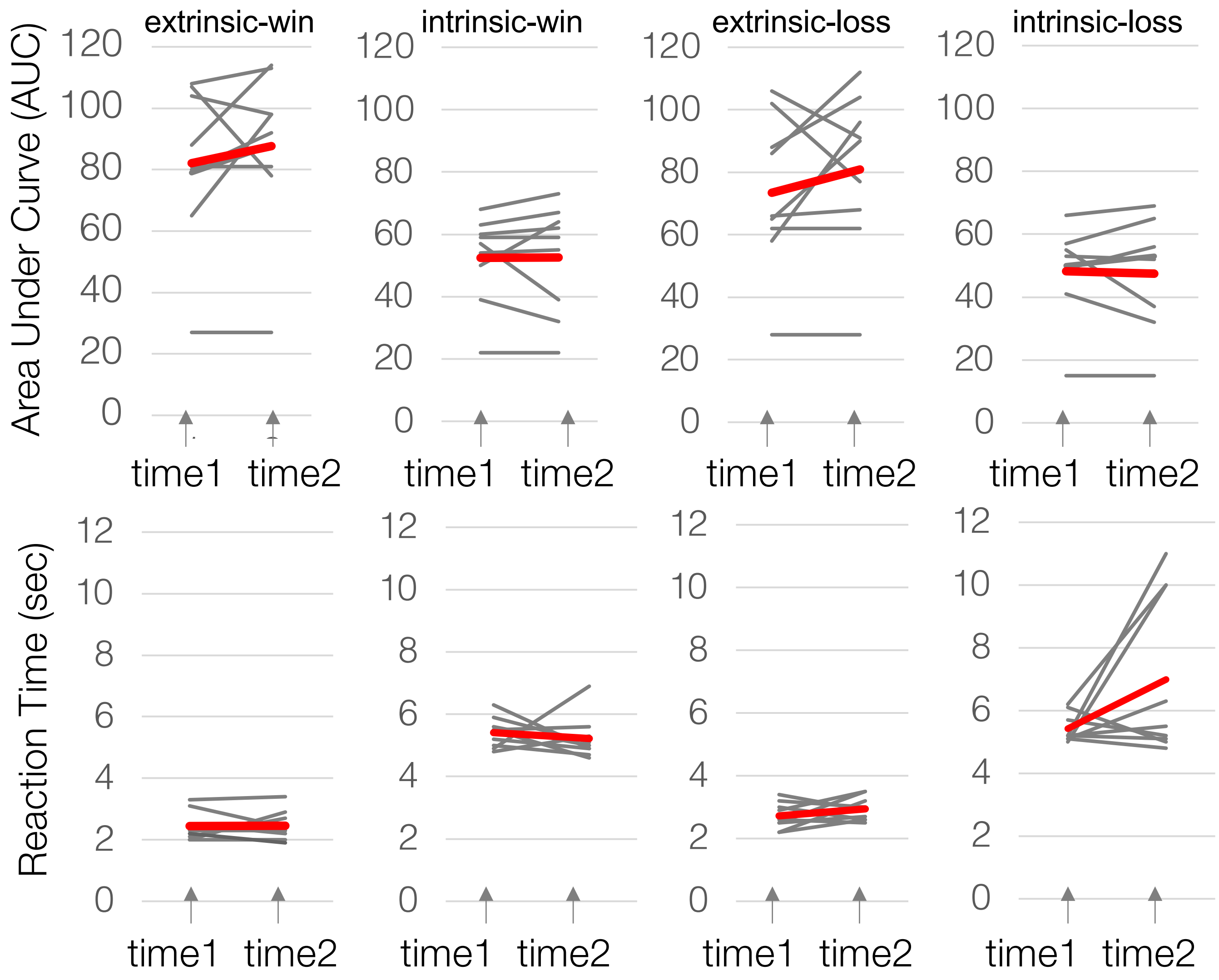

Supplementary Figure 7. Test-retest reliability was assessed in participants (N=10), who completed the task twice. Good stability over time was found for effort-by-reward discount curves (area under the curve) (top-panel) and reaction times (bottom-panel).
